# The Effects of Rhythmic Sensory Cues on the Temporal Dynamics of Human Gait

**DOI:** 10.1371/journal.pone.0043104

**Published:** 2012-08-21

**Authors:** Ervin Sejdić, Yingying Fu, Alison Pak, Jillian A. Fairley, Tom Chau

**Affiliations:** 1 Department of Electrical and Computer Engineering, University of Pittsburgh, Pittsburgh, Pennsylvania, United States of America; 2 Department of Electrical and Computer Engineering, University of Toronto, Toronto, Ontario, Canada; 3 Department of Social and Developmental Psychology, University of Cambridge, Cambridge, United Kingdom; 4 Faculty of Medicine, University of British Columbia, Vancouver, British Columbia, Canada; 5 Bloorview Research Institute, Holland Bloorview Kids Rehabilitation Hospital and the Institute of Biomaterials and Biomedical Engineering, University of Toronto, Toronto, Ontario, Canada; Bielefeld University, Germany

## Abstract

Walking is a complex, rhythmic task performed by the locomotor system. However, natural gait rhythms can be influenced by metronomic auditory stimuli, a phenomenon of particular interest in neurological rehabilitation. In this paper, we examined the effects of aural, visual and tactile rhythmic cues on the temporal dynamics associated with human gait. Data were collected from fifteen healthy adults in two sessions. Each session consisted of five 15-minute trials. In the first trial of each session, participants walked at their preferred walking speed. In subsequent trials, participants were asked to walk to a metronomic beat, provided through visually, aurally, tactile or all three cues (simultaneously and in sync), the pace of which was set to the preferred walking speed of the first trial. Using the collected data, we extracted several parameters including: gait speed, mean stride interval, stride interval variability, scaling exponent and maximum Lyapunov exponent. The extracted parameters showed that rhythmic sensory cues affect the temporal dynamics of human gait. The auditory rhythmic cue had the greatest influence on the gait parameters, while the visual cue had no statistically significant effect on the scaling exponent. These results demonstrate that visual rhythmic cues could be considered as an alternative cueing modality in rehabilitation without concern of adversely altering the statistical persistence of walking.

## Introduction

Walking is a complicated task governed by the hierarchical control of the primary motor cortex, premotor and supplemental motor cortices, basal ganglia, cerebellum, brainstem, spinal pattern generators and feedback from the vestibular system. In particular, the influence of rhythmic sensory cues on walking dynamics is of immense relevance to neurological rehabilitation. On the rehabilitative front, auditory cues have had positive effects on various gait characteristics of patients with Parkinson's disease (PD) [1], stroke [2] and hemiparesis [3]. In patients with PD, visual cues improved stride length, auditory cues improved cadence, while the simultaneous visual and auditory cues did not improve gait more than each cue alone [1]. Stroke patients generally benefited from auditory pacing for both feet [2]. Patients with PD also improved their gait by using visual cues in the form of inverted walking sticks [4]. Interestingly, these results were mirrored in healthy subjects as well. Paltsev and Elner found that auditory stimuli, in comparison to no stimuli, reduced the amount of time required for muscles in healthy human subjects to respond to a given motor command [5]. Additionally, it has been shown that healthy adults can accurately synchronize movements with multisensory cues [6]. Seemingly then, rhythmic sound patterns increase the excitability of spinal motor neurons, reducing the amount of time required for muscles to respond to a given motor command.

The effects of external pacing are not all beneficial. Indeed, metronomic auditory stimuli alters natural neuromuscular rhythms (i.e., fractal dynamics of gait), pushing the scaling exponent, 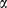, from a value of statistical persistence (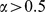) to one of anti-persistence (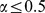) in healthy adults [7], [8]. 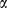 typically ranges from 0.8 to 1.0 in the able-body individuals. However, its value becomes closer to 0.5 due to advanced aging and/or neurological diseases such as PD and Huntington's disease [9], [10], which indicates a time series with weakened statistical persistence. From a clinical point of view, individuals exhibiting lower 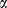 values are more prone to falling [11]. It has been also argued that 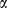 may be a potential indicator how well an individual can adapt to changing walking conditions [12], [13]. Similarly, Terrier and Deriaz showed recently that the maximum Lyapunov exponent, which measures the sensitivity of the system to infinitesimal perturbations (i.e., local dynamic stability) [14], also decreases when a participant is exposed to a rhythmic cue [15].

Therefore, it is unknown whether or not rhythmic cues in other sensory modalities (visual or tactile) induce similar changes on fractal gait dynamics and local dynamic stability. Examples of emerging real-life situations for which such sensory cueing may be relevant are recent efforts to use visual and audio cues in rehabilitation procedures. For example, Frazzitta et al. showed that various cueing modalities can be used to improve gait speed and stride cycle in Parkinsonian patients with freezing [16]. Hence, the goal of the paper is to examine the effect of overground walking to the beat of an auditory, visual and tactile cue on the fractal dynamics and stability of human gait.

We hypothesize that walking to an auditory cue, a visual cue, and/or a tactile cue will negatively impact gait dynamics by displaying a diminished fractal scaling exponent and poorer local dynamics stability as captured by the acceleration of the center of mass. Therefore, to investigate the effect of external rhythmic cues on gait, we are going to explore the temporal dynamics of human gait as measured by the fractal scaling exponent (e.g., [11]) and the maximum Lyapunov exponent (e.g., [14]).

## Materials and Methods

### Subjects

Fifteen healthy, able-bodied subjects (8 females) were recruited from the Bloorview Research Institute, Holland Bloorview Kids Rehabilitation Hospital (Toronto, Ontario, Canada). All participants had normal or corrected-to-normal eyesight and hearing. Subjects that had previous or existing neurological disorder were excluded from the study. None of the participants had an injury or other illnesses that might compromise natural walking.

The mean age of the subjects was 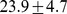 years. The subjects had a mean height and weight of 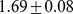 m and 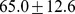 kg, respectively. All participants provided written consent to participate in the study. The study was approved by the Bloorview Research Ethics Board.

### Walking protocol

The study consisted of two sessions, each consisting of five 15-minute trials. During the first trial participants were instructed to walk at their preferred walking speed around an indoor, rectangular path (walkway width 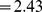 m, perimeter length 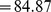 m). The preferred cadence, i.e., the mean number of steps per minute, established the pace at which the participant would be cued in subsequent trials. During the four remaining trials, participants were asked to walk in step to metronomic sensory cues. The cueing modalities were aural, visual and haptic. The intensity of each cueing modality was set such that the participant acknowledged that the cue could be clearly sensed. In three of the trials, participants were cued by a single modality and in one trial, they received simultaneous cues by all three modalities. The cued trials were ordered randomly.

The same protocol was followed for the second session, using a different sequence for the four metronomically cued trials. All participants wore comfortable walking shoes with removable insoles to the sessions. An investigator walked slightly behind the subject during the walking trials. Participants were allowed to take breaks in between trials. After each session was completed, participants were asked if they felt fatigued at any time during the study and responses were noted.

### Apparatus

An ultra-thin, force-sensitive resistor (FSR) (FSR 406, Interlink Electronics) was taped beneath the insole of each subject's right shoe. Each time the FSR made contact with the ground (i.e. when the subject took a step), a change in voltage occurred. These voltages were directly captured by a custom-built datalogger (a programmable R-Engine-A processor board, Tern Inc.). The FSR was connected to the datalogger via a single wire that ran the length of the lateral side of the participant's right leg to his or her back, where the datalogger was housed in a small backpack. Another wire connected a tri-axial accelerometer (MMA7260Q, Freescale Semiconductor Inc.) to the datalogger. The accelerometer was secured to a belt, over the L3 segment of the lumbar spine, close to the standing centre of mass. Accelerations were measured along the three orthogonal axes (anterio-posterior, medio-lateral and vertical). The datalogger collected signals at a rate of 200 Hz and stored the data to a compact flash disk. At the end of each session, data were uploaded to a PC via a serial link for data analysis.

To cue participants, a digital metronome (MA-30, Korg) was attached to the left shoulder strap of the backpack. A custom-built interface was used to connect the metronome to the different cueing modalities. A set of earphones were used to deliver auditory cues. An LED light, used to deliver visual cues, was attached to the end of a rod that was secured to the right side of a bicycle helmet such that it protruded approximately 10 cm in front of the participant's face. Tactile cueing was provided by two single brushed DC pager motors (RPM2, Solarbotics) that vibrated on each beat of the metronome. The motors were enclosed in a pocket on an adjustable glove. All participants wore the glove on their right hand.

### Stride interval dynamics analysis

A probabilistic stride interval extraction algorithm was applied to determine the time series of heel strikes of the same foot (i.e., stride intervals) [17]. The first 30 seconds of data were deleted to avoid “startup” effects (e.g., acceleration to preferred speed). Stride interval variability (SIV) was estimated using the coefficient of variation of stride intervals [18]. Detrended fluctuation analysis (DFA) was performed to quantify stride interval dynamics [10], [11], [19]–[22]. DFA is a technique that was introduced by Peng [23] for the calculation of long range correlations in physiological time series. Briefly, the root-mean square fluctuation of the integrated and detrended time series is calculated at different window sizes. The slope of the log of the fluctuation against the log of window size provides the scaling exponent, 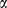. A window size of 16 to N/9 was used for the analysis [24]. The value of the scaling exponent reveals the autocorrelation behavior of the time series. 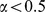 indicates anti-persistence. The series is completely uncorrelated white noise when 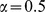. Furthermore, 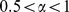 indicates that there is statistical persistence in the time series [11], [23], [24].

### Gait dynamic stability analysis

A growing body of literature has used the maximum Lyapunov exponent to quantify the local dynamic stability of gait [14], [22], [25], [36], with smaller exponents indicative of more locally stable movements. This method determines the sensitivity of the system to infinitesimally small perturbation [14]. In other words, measuring dynamic stability of a system involves measuring the rate of kinematic divergence of a gait cycle trajectory perturbed by naturally occurring disturbances and neuromuscular control errors [26].

The data processing protocol used to determine the Lyapunov exponent follows the approach outlined in [14]. After time delay and embedding dimensions were calculated, the dimensional state vectors were constructed. The time-dependent structure of stride to stride fluctuations was taken into account in the calculation of Lyapunov exponent to provide an index of stability of the gait cycle [22]. Time delay was determined from the autocorrelation function as suggested in [27]. A global false nearest neighbor analysis was used to determine the embedding dimension (e.g., [14]). From the state vectors derived, the Euclidean distance between neighboring trajectories was calculated. The Lyapunov exponents were given by the slope of the average logarithmic divergence of neighboring trajectories in state space. Two exponents were calculated for each walking condition: a short-term Lyapunov exponent (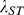) and a long-term Lyapunov exponent (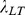). 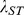 was the slope between the zeroth and first strides, while 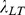 was the slope between the fourth and tenth strides [28].

### Residual examination

In order to understand how accurately participants stepped to different metronomic cues, we calculated residuals, defined as the difference between a heel strike and its nearest metronomic beat (either before or after). The difference is calculated for every other beat, since the metronome pulsed with each step, while heel strike times were only recorded for one foot. The algorithm compensated for the fact that some participants might step ahead of the beat or miss a beat. For example, if a participant missed a step and started following the next metronomic beat, the algorithm would move to the next metronomic beat and calculate the residual from that beat. From the extracted residuals, we calculated the mean and standard deviation for each participant.

### Statistics

The non-parametric Kruskal-Wallis test was used to test for statistical differences in stride interval variability, scaling exponent, mean stride time, gait speed and Lyapunov exponents among the five gait conditions. If significance was found, Mann-Whitney U-tests were performed to compare two conditions at a time using a Bonferroni-adjusted significance level of 0.01 for pairwise comparisons.

## Results

### Stride interval variability and gait parameters

**Figure 1:**
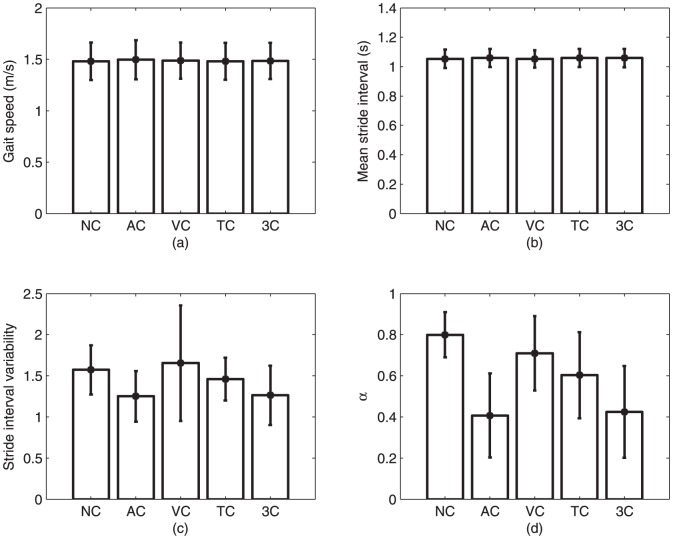
Gait dynamics of participants during the five walking conditions (NC  =  no cue; AC  =  auditory cue; VC  =  visual cue; TC  =  tactile cue; 3C  =  three cues). (a) gait speed; (b) mean stride intervals; (c) stride interval variability; and (d) scaling exponents. Error bars denote standard deviation in each case.

Gait speed (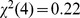, 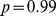) and mean stride interval (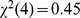, 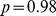) were not statistically different for all five walking conditions (both are depicted in Figure 1). However, stride interval variability (SIV) was statistically different between conditions (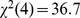, 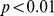). SIV for the trials involving the auditory cue and the threes cues combined exhibited a significant decrease as compared to SIV for visual, tactile and uncued conditions (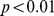). The SIV for the auditory condition was not significantly different from that of the three-cue condition (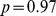), and the SIVs in the visual, tactile and uncued conditions were not significantly different from each other (visual-tactile: 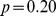, visual-uncued: 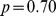, tactile-uncued: 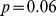). There were no statistically significant differences between session 1 and session 2 for any of the parameters shown in Figure 1.

### Stride interval dynamics

The scaling exponent, 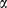, of trials paced with the auditory cue and the three cues was statistically lower than 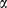 of the non-cued, visually-cued and tactilely-cued conditions (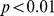). On the other hand, there were no significant differences in 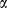 between the non-cued and visually-cued conditions (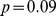), the aurally-cued and three cues conditions (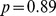), and the visually-cued and tactilely-cued conditions (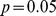), respectively.

### Dynamic stability of gait

**Figure 2:**
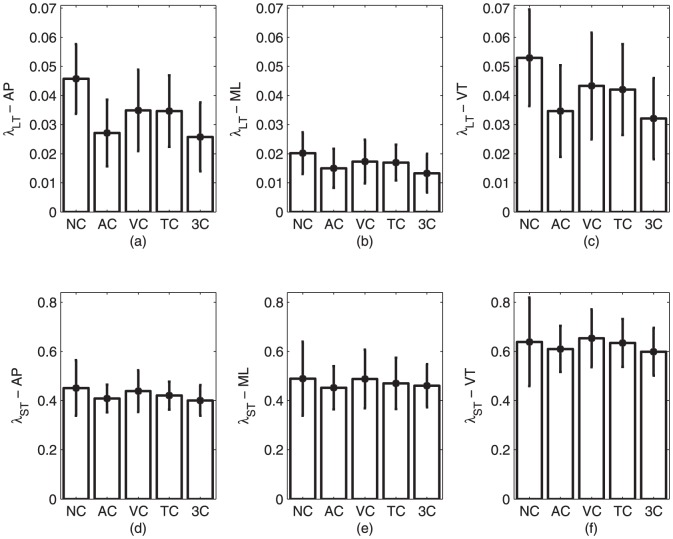
Long and short Lyapunov exponents for the five walking conditions (NC  =  no cue; AC  =  auditory cue; VC  =  visual cue; TC  =  tactile cue; 3C  =  three cues). (a) 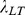 in the AP direction; (b) 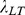 in the ML direction; (c) 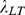 in the VT direction; (d) 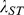 in the AP direction; (e) 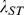 in the ML direction; and (f) 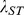 in the VT direction. Error bars denote standard deviation in each case.


Figure 2 represents average Lyapunov exponents, 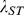 and 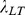, respectively. 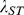 showed no significant differences between any of the conditions in any of the axes (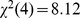 and 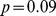 for anterio-posterior (AP); 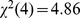 and 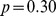 for vertical (VT); 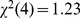 and 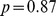 for medio-lateral (ML)).

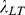
 was statistically different between the five conditions in all three axes (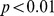). In particular, 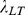 was significantly higher in the uncued condition than in the auditory and three-cued conditions (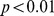 for all three directions). When comparing the non-cued and visually cued conditions, we observed that they were statistically different in the AP direction (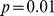). Similarly, the tactilely cued condition was statistically different from the non-cued condition in the AP and VT directions (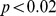). 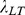 was statistically different between the conditions involving auditory, visual and tactile cues in the AP direction (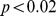). On the other hand, the three-cue condition was statistically different from the visually and tactilely cued conditions in all three directions (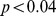).

### Residual examination

**Figure 3:**
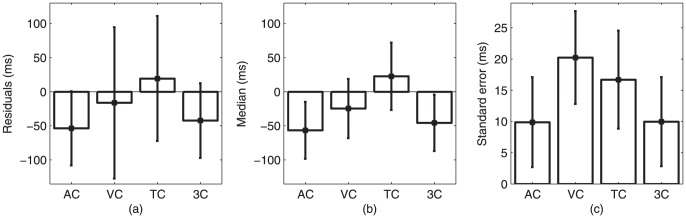
Examination of stepping response, i.e., how well each cue was followed in terms of mean residuals for the four cued conditions (AC  =  auditory cue; VC  =  visual cue; TC  =  tactile cue; 3C  =  three cues). (a) mean residuals (ms); (b) median residuals (ms); and (c) standard error (ms). A negative value means that participants walked ahead of the metronomic beat. Error bars denote standard deviation in each case.

Figure 3 summarizes the results of the residual analysis for all participants. Kruskal-Wallis tests showed that residuals (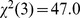 and 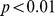), along with the medians (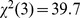 and 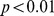) and standard errors (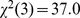 and 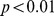), were different among the four conditions. When comparing on a condition-by-condition basis, except for the aurally cued and three cues conditions which were not statistically different (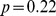), all other conditions were statistically different (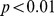 for all). Medians of auditory and three-cued conditions (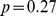), visual and three-cued conditions (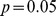) did not statistically differ, while for the rest of the conditions they were statistically different (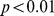 for all). The mean of standard error was also statistically different between the auditory condition and visual and tactile condition (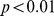 for both); while it was not statistically different for the three-cued trial (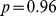). The mean of the standard error for the visual condition were not statistically different from the mean of the tactile condition (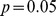), but were statistically different from the three cues condition (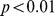).

## Discussion

This quantitative study of healthy subjects' stride interval variability, stride interval dynamics (SID) and dynamic stability while walking to the beat of external cues revealed some interesting results. 1) Walking to the beat of an auditory cue alone or three cues combined significantly reduced a person's SIV; however, walking to visual or tactile cues did not show significant difference in SIV as compared with walking to no stimuli. 2) SID was significantly reduced when walking to an auditory cue, tactile cue, or three cues combined, while it was not significantly changed with a visual cue. 3) Dynamic stability, as calculated from the Lyapunov exponent, increased significantly when walking to an auditory cue or three cues combined, but it was not significantly changed for visually cued or tactilely cued conditions. Common among all results was that, while all external stimuli altered one or more of the measured gait parameters, auditory cues had the greatest affect on one's natural neuromuscular rhythms. We will now discuss these findings in detail.

### Stride interval variability

Our investigation of SIV resulted in two main findings. First, SIV for the auditory and three-cue conditions was significantly lower in comparison to the other conditions. It has been observed that when participants walk to repetitive auditory stimuli, their gait becomes entrained to the rhythmic signals, resulting in more consistent motor unit recruitment patterns [29]. Rhythmic sound patterns increase the excitability of spinal motor neurons via the reticulospinal pathway, reducing the amount of time required for muscles to respond to a given motor command [1], and thus decreasing the possibility for variation. Our research has also found that walking to three simultaneous cues resulted in a similar reduction in SIV as compared to walking to an auditory cue alone. One possible explanation is that the participants only followed the auditory cue, and completely ignored the other simultaneous cues. The second possible explanation that the auditory cue dominated over other cues, since the participants indicated that they generally followed all three cues when simultaneously presented. However, we did not measure the relative perceived weights of individual inputs. Therefore, the current experiment was not designed to differentiate between the two listed possibilities. Future experiments considering multisensory inputs should attempt to determine the relative weight of the individual inputs, since previous experiments considering multisensory cues found that the auditory cues had a prevailing effect over simultaneously administered visual or tactile modalities (e.g., [30], [31], [32]).

Second, the results also showed that SIV did not differ among conditions involving no cues, a visual cue or a tactile cue. Similar findings for the visual cue were presented in [33], [34]. The main reason for unaltered SIV during visual cueing is the fact that healthy adults are able to adjust their walking patterns flexibly using an external visual rhythm without changing walking speed [34], which was demonstrated by our results as well. For the tactile cue, our results follow similar trends as those observed in [45], [36] (i.e., we observed a decrease in the mean SIV value during tactile cueing). However, they failed to reach significance in our case (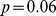). A potential reason for this observed difference between our results and the previous findings could be in the position of a tactile cue. We provided rhythmic vibrations to the back of a participant's hand, while an insole with a vibratory device and a wristband-based vibratory device were used in [35] and [36], respectively.

### Stride interval dynamics

The strength of stride interval dynamics (i.e., the scaling exponent, 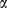) has been used as a measure of gait unsteadiness (e.g., [20]). A reduced scaling exponent has been found for patients with gait unsteadiness, such as Parkinson's disease and Huntington's disease [37]. External auditory pacing can override the naturally occurring statistical persistence [7]. Our results follow previously reported trends. In particular, we found that different cuing modalities influenced a person's gait dynamics to different degrees. The results showed that the aurally cued and the triple cues conditions reduced the scaling exponent the most, followed by tactilely cued and visually cued conditions. Jahn et al. [38] have speculated that during metronomic walking an auditory stimulus traverses the central auditory system and relays the processed information to a supraspinal locomotor “clock”, possibly the vermis (which is known to integrate incoming proprioceptive, exteroceptive, visual and vestibular afferent information). As discussed earlier, research has shown that, compared to visual rhythms, auditory rhythms elicit a greater involuntary, automatic response from the neural system responsible for sensorimotor coordination [39]. Thus, in this study, the auditory stimuli may have more effectively overridden the internal clock, resulting in a greater influence on gait.

Our analysis of stride dynamics also showed that the visual cue did not significantly alter the scaling exponent in comparison to the non-cued trial. These results are similar to the results of a recent study which showed that visual cues did not alter step amplitude [33]. The fact that the visual cue did not alter the gait dynamics in healthy subjects could have possible implications in rehabilitation, since it has already been shown that visual cues can improve stride length [1]. Furthermore, it has been shown that external rhythmic cueing can help patients with Parkinson's disease (e.g. [34], [40]), presumably by compensating for basal ganglia disease causing an inability to internally generate rhythmic movements [34]. A possible explanation for the absence of a negative effect during visually cued conditions, which was supported by subjective comments from the participants, is that their focus was drawn away from the possibly destabilizing visual stimuli in the environment, allowing gait to proceed undisturbed under the guidance of the visual rhythm [34].

When considering the stride dynamics for the tactilely cued trial, it was interesting to note that this trial did not statistically differ from the visually cued trial (though it had a very low 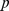 value), but it was statistically different from the uncued trial. In particular, the tactile cues weakened the statistical persistence (i.e., decrease 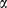) in comparison to the non-cued trial. Nevertheless, these cues did not have as adverse an effect on participants' natural gait rhythms as auditory pacing. As mentioned previously, rhythmic tactile cues lower stride frequency, and increase stride length [36]. Also, it has been shown that tactile cues positively affect the human postural control system (i.e., head and body sway) [41]. Hence, based on the results presented in this study and similar studies (e.g., [36], [41]), we anticipate that tactile cues could have positive effect on gait as well. However, further studies are necessary to fully examine these effects more closely, such as, how the location of the cue on the body affects the stride dynamics.

### Dynamic stability of gait

Our results showed that the rhythmic cues considered in this paper had no immediate effects on the next stride (i.e., they did not alter the dynamic stability over a short time period). In particular, 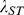 was not statistically different between all trials in any considered direction. On the other hand, the long-term dynamic stability, as assessed by 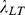, was altered with certain cues. Walking to an auditory cue, or walking to three cues combined, improved dynamic stability as indicated by the decrease in the long term Lyapunov exponent in all three axes. In particular, the auditory cue may have had a greater effect than the other cuing modalities for the reasons it impacted in SIV and SID.

We also noticed a relationship between scaling and Lyapunov exponents, such that a decrease in LE corresponded to a decrease in the scaling exponent and vice versa. This result was also observed in research done by Jordan et al. [22]. Specifically, Jordan et al. found that lower stability, as measured by a higher LE, was associated with a higher scaling exponent, and hence more persistent stride-to-stride fluctuations [22]. They suggested that increased scaling exponents may have occurred because, as gait becomes more unstable, the influence of central control is increased [22]. Previous research has attributed the strength of the scaling exponent to the supraspinal locomotor clock [7], and so an increase in the supraspinal control may cause the scaling exponent to increase.

### Residual examination

While the residuals were statistical different between conditions, the magnitude of the residuals for different cueing approaches was at least an order of magnitude smaller than the average stride interval. Hence, these differences in residuals could not play a significant role in statistical differences observed in the scaling exponent values. Furthermore, our results agree with previous findings which have demonstrated a dominance of one cuing modality over another (for example, visual or auditory) when multiple temporal cues are presented [39]. Specifically, we observed a dominance of auditory cuing over all other cues, since residuals were not statistically different between the conditions involving an auditory cue and three cues.

We also found that the variability of residuals was smaller for the trials involving the auditory cue. These results are in accordance with [39] which showed that there was a greater variability of asynchronies in response when presented with visual stimuli compared to auditory stimuli. These results suggest that auditory rhythms are more strongly coupled to the motor system than to visual rhythms [42]. Another study showed that even when attention was biased as much as possible toward visual cues, participants still showed involuntary responses attributed about 70% to auditory and 30% to visual stimuli; reflecting a clear auditory dominance [39]. Furthermore, Bresciani previously found that in non-spatial tasks, task-irrelevant auditory stimuli often biased visual and tactile perceptual estimates [31]. For example, single visual flashes or single tactile taps were perceived as multiple flashes or taps when multiple auditory beeps were presented simultaneously. In other words, when the CNS was presented with a pool of multimodal sensory signals, it tended to automatically integrate auditory and tactile signals [31]. However, the current experiment was not designed to assess the relative strength of each simultaneous cueing modality and future studies should consider this matter more closely.

### Remarks

We should also comment on the interesting findings of trials involving the auditory cue. In particular, we found that SIV decreased while walking to an auditory cue, implying more stable walking. In contrast, analysis of the scaling exponent showed that auditory cues significantly altered one's natural stepping rhythm. Thus, walking to an external beat may impose unnatural neuromuscular rhythms on the otherwise highly fractal dynamics of human gait, resulting in a loss of functional adaptability (for example, impaired dynamical balance and responsiveness to perturbations) [37]. Stride interval dynamics may be more sensitive to smaller perturbations than those naturally occurring from stride to stride, the latter which only focuses on a participant's average stride. To this end, Herman et al. found that the scaling exponent was able to differentiate fallers from non-fallers among patients with “higher-level” gait disorder, while stride interval variability failed to do so [20].

## Conclusions

In this paper, we examined the effects of various external rhythmic cues on human gait. In particular, we considered auditory, visual and tactile rhythmic cues. The results showed that all of these different cues affected the measured variables to a certain extent, including stride interval variability, the scaling exponent and the Lyapunov exponent. In particular, the aurally-cued condition and the three cues condition decreased stride interval variability. Furthermore, the cues decreases the value of fractal scaling exponents in the observed stride interval time series. The auditory cue had the strongest negative impact on persistence, while the visual cue was not statistically different from walks with no stimuli. Also, the aurally-cued condition and triple cue condition produced smaller long-term Lyapunov exponents, suggesting dynamically more stable systems. The current results also suggested that future studies should measure the relative weights of individual cues during multisensory cueing.
